# Ankfy1 Is Involved in the Maintenance of Cerebellar Purkinje Cells

**DOI:** 10.3389/fncel.2021.648801

**Published:** 2021-03-16

**Authors:** Liansheng Chang, Shahid Hussain Soomro, Hongfeng Zhang, Hui Fu

**Affiliations:** ^1^Department of Anatomy, School of Basic Medical Sciences, Wuhan University, Wuhan, China; ^2^Department of Pathology, Tongji Medical College, The Central Hospital of Wuhan, Huazhong University of Science and Technology, Wuhan, China

**Keywords:** Ankfy1, cerebellum, Purkinje cells, degeneration, ataxia

## Abstract

Purkinje cells are critical for the function of cerebellum. The degeneration of Purkinje cells leads to defects in motion control. We have found that Purkinje cells specifically express Ankfy1 protein during development and in adult. This protein seems to play minor functions during development as Ankfy1 knockout mice appear normal till adult. However, at 9-month-old, knockout mice showed abnormal cerebellum with reduced vermis size and developed defective motor function. Further investigation demonstrated that the cerebellum of the mutant mouse has lost most of its Purkinje cells, while other cerebellar cells remained largely normal. Our data suggested that the *Ankfy1* might be important for the maintenance of cerebellar Purkinje cells.

## Introduction

The Purkinje cells (PCs) is important for the function of cerebellum. As the only output cell type in cerebellar cortex, the PC is a highly specialized cell type with a big pear-shaped cell body and a large dendritic arbor. The soma of PC is located between the outer molecular layer and the inner granular layer, forming the PC layer. Functionally, PCs are critical for motor control, involved in the planning and fine-tuning of movement and coordination (Medina, 2011). Clinically, the loss of PCs would result in cerebellar ataxia. For now, at least 49 subtypes of spinocerebellar ataxias (SCA) have been reported with the common feature of neurodegenerative PC loss (Huang and Verbeek, 2019).

We have found a membrane-bound protein, Ankfy1, specifically expressed in mouse PCs. Previously, we reported that the knockout mice showed no obvious phenotype during development and in young adults (2 months) if they survived early embryonic stages, as the homozygotes experienced embryonic lethality in pure genetic background (Weng et al., 2016). In the mixed background mouse line, we found the knockout mice gradually developed motor dysfunction, which is quite obvious at 9-month-old. A series of experiments were performed and revealed that the knockout mice underwent cerebellar PC degeneration as the mice aged.

## Materials and Methods

### Animals

The generation of Ankfy1 knockout mice has been previous reported (Weng et al., 2016). Briefly, the chimera mouse was ordered from the Mutant Mouse Resource and Research Center (MMRRC, UC Davis, CA, USA). The mice were housed in the Center for Animal Experiments/Animal Biosafety Level-III Laboratory in Wuhan University in accordance with the National Institutes of Health Guide for the Care and Use of Laboratory Animals (National Research Council Publication, 1996 edition) and the protocol was approved by the Institutional Animal Care and Use Committee of Wuhan University.

Only male mouse was used with at least three animals for any group throughout the study. All the Ankfy1 knockout mice in this study are of generation 3 or 4 with C57BL/6 mice.

### *In situ* Hybridization

The *Ankfy1* probe was made by reverse transcription-polymerase chain reaction (PCR) from an embryonic day 13.5 (E13.5) mouse embryo. The primer sequences were CAC AAG TTT GTC TTG GCC GC and-GGT CTA ATG GGT CTC CTG GG. The PCR product was 741 bp in size and cloned into a pSport1 plasmid. *In situ* hybridization was performed as previously described (Fu et al., 2009).

### Immunostaining

The immunostaining procedure was performed as previously described (Fu et al., 2009). Briefly, the slides were washed three times with PBS, 10 min each. Afterwards, slides were blocked by 5% normal goat serum at room temperature for 1 h. Then, primary antibodies were added and incubated at 4°C overnight. In the second day, slides were washed in PBS three times, 10 min each, followed by incubation with secondary antibodies (Jackson ImmunoResearch, PA, USA) in PBS for 1 h. The slides were washed three more times in PBS. Pictures were taken using a Nikon Eclipse Ni fluorescent microscope (Nikon, Tokyo, Japan).

For the *in situ* hybridization combination with Immunostaining experiments, the immunostaining procedures are performed after the *in situ* hybridization. The images of the *in situ* hybridization were pseudo-colored by PhotoShop software and then combined with the immunofluorescence color.

The antibodies used in this study included: anti-calbindin-D-28 (1:400, C9848, sigma), anti-parvalbumin (1:250, ab181086, Abcam), anti-Zic (1:500, from Dr. Segal's lab, DFCI, Boston, MA, USA), anti-GFAP (1:100, ab10062, Abcam).

### Rotarod Experiments

Mice were tested using a rotarod apparatus (YLS-4C, Ruanlong Tech., Shanghai) to evaluate their motor performance. The protocol consists of 3 days of training at a constant speed (10 rpm) for 10 min in three trials, with a 10-min interval between each trial. On the fourth day, the animals were subjected to four trials on an accelerating rod (4–30 rpm, 5 min) with a 10-min interval between each trial. Rotarod performance was quantified by recording the latency to fall off the apparatus.

## Results

### Ankfy1 Is Expressed in Purkinje Cells

**Figure 1:**
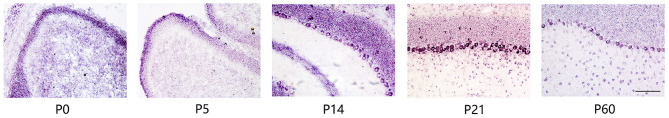
*In situ* hybridization results showing *Ankfy1* mRNA expression in the murine cerebellum from postnatal day 0 (P0) to P60. Scale bars: 100 μm.

**Figure 2:**
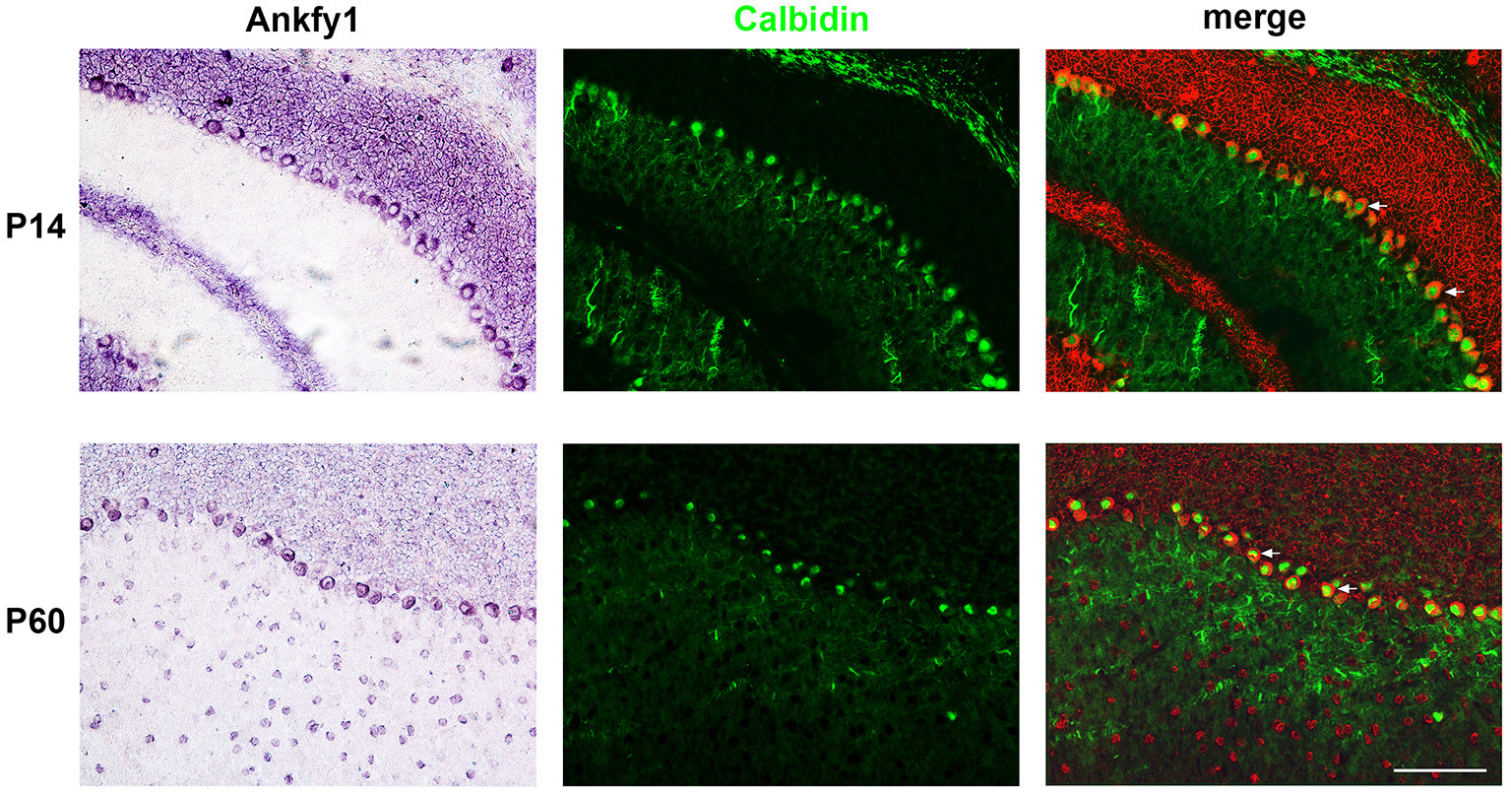
*In situ* hybridization results of *Ankfy1* combined with immunostaining of calbindin on the murine cerebellum of P14 and P60. The *In situ* hybridization signals are psudocolored in red. The immunostaining signals are showed here in green color. The arrows showed the double stained Purkinje cells. Scale bar: 100 μm.

We studied the expression of *Ankfy1* in the mouse cerebellum. *In situ* hybridization experiments revealed that *Ankfy1* mRNA appears in the outer edge of the developing cerebellum at early stages (P0, P5) (Figure 1). At P14, *Ankfy1* mRNA is expressed in the newly appeared PCs (Figure 1), which was confirmed by double staining of calbindin, a specific marker for the PCs (Figure 2). At later developing stages such as P21 (Figure 1) and P30 (data not shown), *Ankfy1* continues to be expressed in PCs. In adults (P60), mouse PCs keep on expressing *Ankfy1* (Figures 1, 2).

### Loss of Ankfy1 Leads to Morphological Change and PC Degeneration in Cerebellum at 9 Months Old

**Figure 3:**
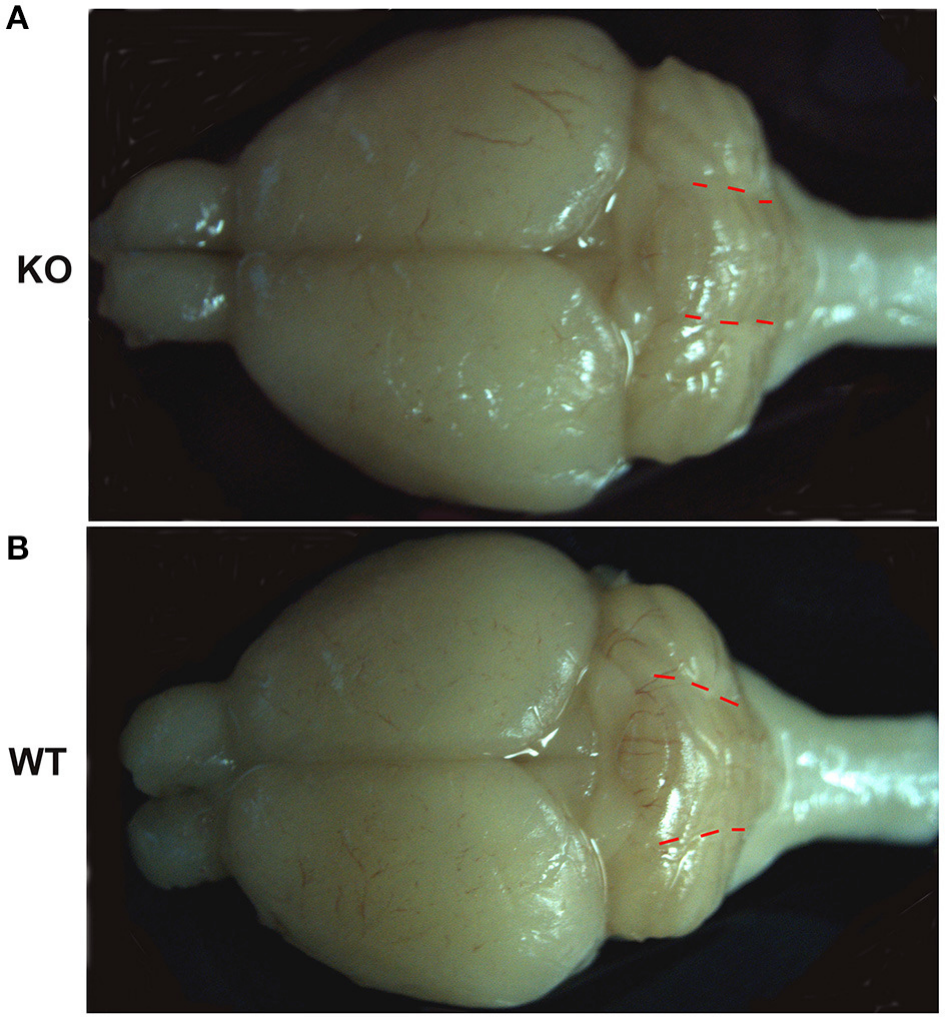
The representative whole brain pictures of Ankfy1 knockout mice **(A)** and the wildtype littermates **(B)** at 9 months. The red dotted lines demarcate the border between the cerebellar vermis and the cerebellar hemisphere.

Our previous work reported no observed defects in young ankfy1 knockout mice till 2 months old. Histopathology study of major organs in mutant mice revealed no obvious abnormity including cerebellum. The shape and number of PCs showed no difference between homozygotes and wildtype littermates at 2-month-old (Weng et al., 2016). However, in this study we found that the cerebellum in Ankfy1 knockout mice is morphologically different from that in the wildtype mice at 9 months old. The cerebellar vermis of knockout mice is obviously reduced compared with that of wildtype mice. The morphology change is more recognizable when the ratio of the vermis width to the hemisphere width is measured. The width of cerebellar vermis in the knockout mouse is about the same as that of the cerebellar hemisphere, while in wildtype mice, the width of vermis is significantly bigger than that of the cerebellar hemisphere (Figure 3). These results suggested that Ankfy1 may not important for cerebellar development but critical for its maintenance.

**Figure 4:**
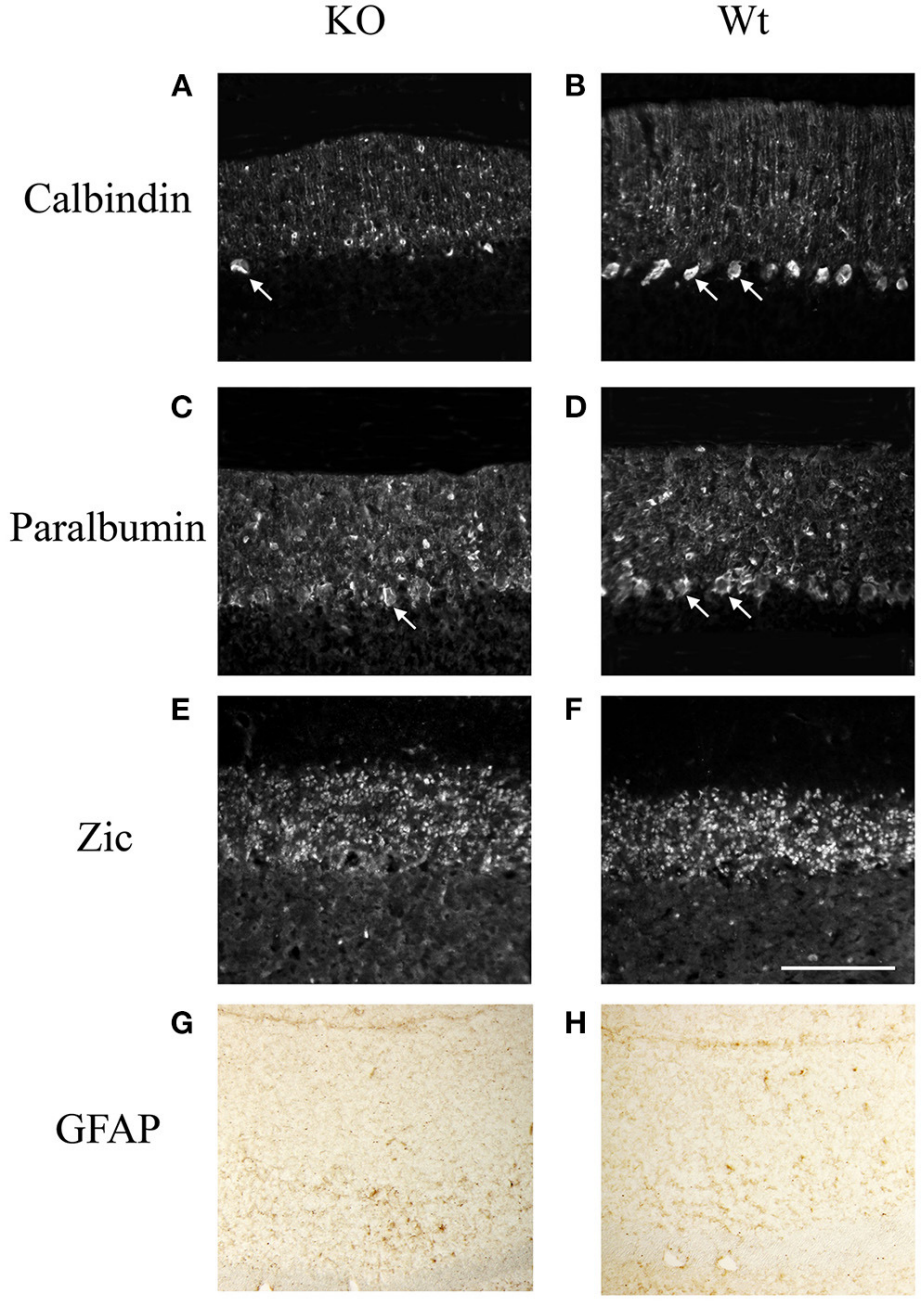
The immunofluorescent staining showing the expression of calbindin **(A,B)**, parvalbumin **(C,D)**, zic **(E,F)** and Immunohistochemistry staining of GFAP protein **(G,H)** in Ankfy1 Knockout (KO) and wildtype littermate (WT) mice at 9 months. The arrows shows the Purkinje cells. Scale bar: 100 μm.

Further study showed that the cerebellum of knockout mouse underwent cellular degeneration at an old age. At 9-month-old, the number of calbindin positive Purkinje cells has greatly decreased in the mutant mice compared with that in the wildtype littermates (Figure 4). We have also checked other neural cell types. Parvalbumin is a calcium-binding protein, which is expressed mostly in Basket and stellate cells in molecule layers and Purkinje cells (Bastianelli, 2003). Results showed that parvalbumin positive cells in the molecular layer are the same between knockout mice and wildtype littermates, while most of the parvalbumin positive cells in the Purkinje cell layer are lost in the knockout mice (Figure 4). We also examined the expression of Zic proteins, which are expressed in granule cells in the granular layer (Borghesani et al., 2002). No difference of Zic expression is found between the knockout mice and the wildtype littermates (Figure 4). We also checked with astrocyte marker (GFAP) and found no obvious changes between the wildtype and the KO mice (Figure 4). Results suggested that Ankfy1 knockout mice underwent Purkinje cell degeneration without significant gliosis.

### Loss of Ankfy1 Causes Defects in Motor Function

**Figure 5:**
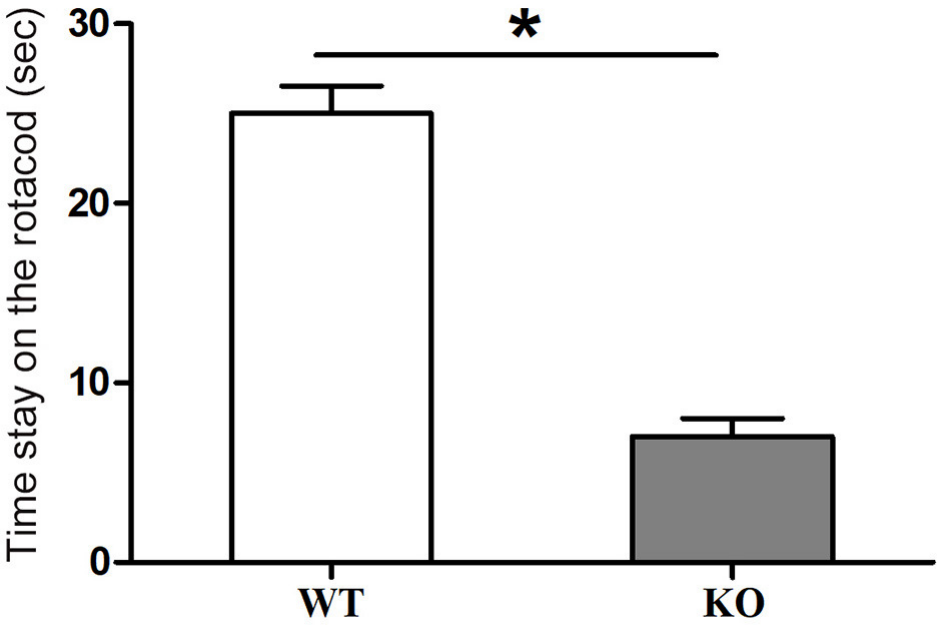
Rotarod experiment results showing motor performance of Ankfy1 Knockout (KO) and wildtype littermate (WT) mice at 9 months. Differences between WT (*n* = 3) and KO mice (*n* = 3) were statistically significant (*p < 0.05). The data represent means ± SEM.

At 9 months old, the homozygotes showed some movement disorders. The rotarod experiment was performed. The results showed that mutant mice stayed on the rolling bar for a much shorter time than the wildtype littermates. We used an acceleration mode for the rotacod test. The results also indicated that the mutant mice fell off the apparatus at much lower rolling speeds than the wildtype littermates (Figure 5). From this experiment, we concluded that Ankfy1 mutant mice experienced some motor dysfunction.

## Discussion

In this study, we found that Ankfy1 is expressed early in the developing cerebellum and quite specifically expressed in developing PCs. However, the Ankfy1 knockout mice showed no obvious neural defects during development till young adult (Weng et al., 2016). As the mice age, the Ankfy1 knockout mouse gradually develops motor dysfunction. At the age of 9 months, the knockout mice showed obvious motor defects which can be readily detected by rotarod experiments. Concurrently, the knockout mice demonstrated some morphological changes of the brain especially the cerebellum. The cerebellar vermis size of the Ankfy1 knockout mouse is much reduced compared with that of the wildtype littermate (Figure 3). Immunohistochemistry study revealed that the Ankfy1 knockout mice suffered from dramatic loss of cerebellar PCs while other types of cerebellar cells remain grossly normal at 9-month-old. Our data suggested that Ankfy1 plays important roles for PC maintenance and the loss of ankfy1 gene leads to PC degeneration and cerebellar ataxia.

### Function of Ankfy1 in the Development and Maintenance of Neural Cells

Ankfy1 is expressed very early in the developing central nervous system. During early development, Ankfy1 is expressed in the neural stem cells. It appears in the ventricular zone of the spinal cord and forebrain where the neural stem cell/progenitor cell located (Weng et al., 2016). In this study, we found it is also expressed in the outer edge of the cerebellum at early stages where the germinal zone located. However, our previous study revealed that Ankfy1 does not play an active role for neural development. The development process seems normal for neural stem cells/progenitors, neurons and glia in the ankfy1 knockout mice. The knockout mice develop normally if they survive early embryonic stages and showed no obvious defects in their young adulthood. Histopathological examination and behavior study revealed no abnormality in the knockout mice at 2-month-old (Weng et al., 2016). However, it has been noticed that Ankfy1 knockout mice died in early embryonic stages in pure genetic background and the reason has been speculated to be functional roles of Ankfy1 in extraembryonic tissues (Weng et al., 2016).

In adults, Ankfy1 continued to be expressed in some neurons such as Purkinje cells in the cerebellum and neurons in spinal cords. Gradually, the knockout mice develop motor defects, which become noticeable at the age of 6 months (data not shown). At 9-month-old, rotarod experiments showed that the motor function of the knockout mice has been greatly compromised compared to that of the control mice. This motor defect correlates well with the drastic loss of cerebellar Purkinje cells in the Ankfy1 knockout mice, suggesting that Ankfy1 knockout mice develop cerebellar PC degeneration which leads to motor defects. Other cell types in the cerebellum of mutant mice exhibited no obvious defects. It is an interesting observation that the cerebellar vermis of the knockout mice decrease its size at an old age (9 months). Overall, our data support that Ankfy1 is critical for the maintenance of PCs.

We have noticed a previous study claiming Ankfy1 is important for PC development (Ding et al., 2017). We have not observed any developmental defects in Ankfy1 knockout mice. It is interesting to note that all the data from this paper are based on the work on Ankfy1 heterozygotes. We have showed that in pure background Ankfy1 knockout mice had extraembryonic tissue defects and died before E13.5 (Weng et al., 2016). Since this work contained no data from homozygotes, we suspect that their mutant mice are in pure genetic background. Their observation could be the phenotype caused by defective extraembryonic tissues.

### Possible Mechanism of Ankfy1 Function on Neuronal Degeneration

Ankfy1 protein is a membrane protein. It can bind to small GTPase RAB5 (Ito et al., 1999). Studies showed that it involved in intracellular endosome/vesicle trafficking (Schnatwinkel et al., 2004; Zhang et al., 2012). It is also part of PDGF signaling pathway. Loss of Ankfy1 could cause a small decrease (30%) of the PDGF signaling (Schmees et al., 2012; Nehru et al., 2013). This small shortage of growth factor signaling could be detrimental for neuronal cells. Studies have shown that one of the reasons for neurodegenerative diseases is the decreased levels of growth factors (Zuccato and Cattaneo, 2009; Bartus and Johnson, 2017). It is likely that Ankfy1 loss leads to a small disturbance PDGF signaling which would significantly affect the PC survival. It is worth noting a previous study on Ascl2 protein. Ascl2 is also a RAB5 binding protein involved in endocytosis. Als2-null mice showed a slowly progressive loss of cerebellar Purkinje cells without obvious developmental abnormalities, which is very similar to the phenotype of Ankfy1 knockout mice. This PC loss may be caused by defective EGF signaling as analysis showed significantly smaller-sized EGF-positive endosomes in Als2-null fibroblasts (Hadano et al., 2006).

### Human Diseases Implication

Clinically, hereditary neurodegenerative disorders caused by pervasive Purkinje cell loss in the cerebellum are named as spinocerebellar ataxias (SCA). For now, there are 49 subtypes of SCA, among which 38 causative genes have been identified (Huang and Verbeek, 2019). However, *Ankfy1* gene is not one of them. In year 2007, a genetic study suspected three novel genes as the causative genes in ataxia families. *Ankfy1* is one of them. Further DNA sequencing found no abnormalities in coding regions in all three suspected genes (Bouslam et al., 2007). It is possible that Ankfy1 was dysregulated and altered levels of Ankfy1 led to ataxia in cases of this study.

Over all, we found that Ankfy1 is expressed in developing and mature PCs in cerebellum. The deficiency of Ankfy1 leads to the degeneration of PCs, but not the developmental defects. Since Ankfy1 protein is a membrane protein involved in growth factor signaling, the loss of Ankfy1 would attenuate the growth factor signaling which is critical for PC survival. Further study on Ankfy1 would be helpful to understand the mechanism of PC degeneration and help to develop therapies for SCA diseases.

## Data Availability Statement

The original contributions presented in the study are included in the article/supplementary materials, further inquiries can be directed to the corresponding authors.

## Ethics Statement

The animal study was reviewed and approved by the Institutional Animal Care and Use Committee of Wuhan University.

## Author Contributions

HF designed the project, guided the project, and revised the manuscript. HZ helped to design the project, guided the project all through, and revised the manuscript. LC and SS did the experiments and wrote the draft. All authors contributed to the article and approved the submitted version.

## Conflict of Interest

The authors declare that the research was conducted in the absence of any commercial or financial relationships that could be construed as a potential conflict of interest.
